# Inflammatory Mediators of Endothelial Dysfunction

**DOI:** 10.3390/life13061420

**Published:** 2023-06-20

**Authors:** Eirini Dri, Evangelos Lampas, George Lazaros, Emilia Lazarou, Panagiotis Theofilis, Costas Tsioufis, Dimitris Tousoulis

**Affiliations:** 1 1st Department of Cardiology, Hippokration General Hospital, Kapodistrian University of Athens Medical School, Vas. Sofias 114, 11528 Athens, Greece; 2 Department of Cardiology, Konstantopouleio General Hospital, 14233 Athens, Greece

**Keywords:** endothelial dysfunction, atherosclerosis, inflammatory modulators, NLRP3 inflammasome, H2S gasotransmitter

## Abstract

Endothelial dysfunction (ED) is characterized by imbalanced vasodilation and vasoconstriction, elevated reactive oxygen species (ROS), and inflammatory factors, as well as deficiency of nitric oxide (NO) bioavailability. It has been reported that the maintenance of endothelial cell integrity serves a significant role in human health and disease due to the involvement of the endothelium in several processes, such as regulation of vascular tone, regulation of hemostasis and thrombosis, cell adhesion, smooth muscle cell proliferation, and vascular inflammation. Inflammatory modulators/biomarkers, such as IL-1α, IL-1β, IL-6, IL-12, IL-15, IL-18, and tumor necrosis factor α, or alternative anti-inflammatory cytokine IL-10, and adhesion molecules (ICAM-1, VCAM-1), involved in atherosclerosis progression have been shown to predict cardiovascular diseases. Furthermore, several signaling pathways, such as NLRP3 inflammasome, that are associated with the inflammatory response and the disrupted H2S bioavailability are postulated to be new indicators for endothelial cell inflammation and its associated endothelial dysfunction. In this review, we summarize the knowledge of a plethora of reviews, research articles, and clinical trials concerning the key inflammatory modulators and signaling pathways in atherosclerosis due to endothelial dysfunction.

## 1. Introduction

The human endothelium is a vast organ that can produce a plethora of molecules, often with opposing effects that contribute to maintaining homeostasis, including vasodilatory and vasoconstrictive, procoagulant and anticoagulant, inflammatory and anti-inflammatory, fibrinolytic and antifibrinolytic, and oxidant and antioxidant substances [1]. The healthy endothelium is responsible for six critical functions in regulating vascular homeostasis: (1) modulation of vascular permeability, (2) modulation of vasomotor tone, (3) modulation of coagulation homeostasis, (4) regulation of inflammation and immunity, (5) regulation of cell growth, and [2] oxidation of LDL cholesterol [3]. These functions are mediated by numerous factors. In this review, we will focus on the recent literature concerning the role of inflammatory mediators in endothelial dysfunction and, consequently, in atherosclerosis and cardiovascular diseases.

Clinical and experimental studies, over the last two decades, have shown that atherosclerosis is a low-grade, sterile, inflammatory disease [4,5]. The development and progression of cardiovascular disease (CVD), ranging from endothelial dysfunction to clinical syndromes, is strongly associated with systemin and local inflammation [5,6,7,8,9]. In addition to the traditional risk factors, several inflammatory biomarkers have been identified to predict CVD [10,11]. Various acute and chronic conditions, including psychological stress, autoimmune disease, microbial and viral infections, and aging, as well as traditional risk factors, can lead to endothelial damage and dysfunction [12,13,14]. This, in turn, triggers a low-grade inflammatory response in blood vessels, leading to the progression of atherosclerosis [15]. Consequently, inflammation is a common mechanism linking traditional and emerging cardiovascular risk factors (CVRF) to the development of atherosclerosis, which can result in coronary artery disease (CAD), large artery thrombotic stroke, and cerebral aneurysms [16].

### Endothelial Dysfunction and Inflammation

An imbalance in the production or bioavailability of endothelium-derived NO results in a decreased vasodilator response and a prothrombotic and proinflammatory endothelium, causing endothelial dysfunction. During the inflammatory process induced by different risk factors, such as hypertension, oxidized LDL (oxLDL), and diabetes, there is an increase in the production of pro- and anti-inflammatory mediators, such as cytokines, such as interleukin-1 (IL-1), IL-6, IL-18, tumor necrosis factor (TNF-α) and C-reactive protein (CRP), PTX3 (inflammation biomarker, which is secreted locally by monocytes/macrophages, ECs, VSMCs, and other cell types, reaching a peak faster than CRP (Moore, 2011 #2282)), that create the endothelial proinflammatory phenotype characterized by an increase in E-selectin, vascular cell and intracellular adhesion molecule-1 (VCAM-1 and ICAM-1) expression, and monocyte chemoattractant protein-1 (MCP-1) in the endothelial cell [17,18,19,20].

Several CVRFs reduce the bioavailability of NO and increase permeability to macromolecules at arterial side branches with disturbed blood flow and low wall shear stress. This procedure leads to subendothelial lipid retention, oxidation, and aggregation. The next step is phagocytosis by resident macrophages and dendritic cells, which contribute to foam cell formation and secrete several adhesion molecules, such as VCAM-1, ICAM-1, and E-selectin on the endothelial surface, triggering the recruitment of circulating monocytes. The recruited monocytes differentiate into macrophages in the subendothelial space and then are polarized in response to their microenvironment, adopting different functional phenotypes [21]. The activation of macrophages into proinflammatory M1 or anti-inflammatory M2 macrophages is initiated by T lymphocytes. The former type of macrophages releases proinflammatory cytokines (e.g., interleukin (IL)-1α, IL-1β, IL-6, IL-12, IL-15, IL-18, and TNF-α), which are known to contribute to the progression of atherosclerosis, while the latter type releases anti-inflammatory cytokines (e.g., IL-10 and transforming growth factor (TGF-β), which play a critical role in inflammation resolution and plaque healing [22,23]. C-reactive protein (CRP), an established biomarker of cardiovascular risk, is produced in the liver under the control of IL-1β, IL-6, and IL-12 [24]. Although macrophages are the primary source of cytokines, other cells, including lymphocytes, endothelial cells, and polymorphonuclear leukocytes, also contribute to their production. Most immune system components produce either proinflammatory or anti-inflammatory soluble factors and cells, depending on the inflammatory environment. An imbalance between the proinflammatory and anti-inflammatory activities of immune cells drives the progression of atherosclerotic plaque [18]. Overloaded foam cells will eventually undergo apoptosis or necrosis, forming a necrotic core [25]. A fibrous cap, which acts as a barrier in more developed lesions, is composed of vascular smooth muscle cells (VSMCs) that migrate from the media into the intimal layer of the vasculature. VSMCs primarily occupy the area just beneath the endothelial cells’ (ECs) lining and are crucial in stabilizing the plaque. In the context of atherosclerosis, VSMCs shift from a contractile state to a synthetic one, which is important in preserving vascular tone and function. This transformation enables VSMCs to facilitate migration, proliferation, and the production of the extracellular matrix (ECM) that promotes plaque stability [26]. Vascular remodeling could be caused by the interplay of EC dysfunction, VSMC migration and proliferation, foam cell formation, and enhanced secretion of inflammatory mediators, such as cytokines.

Furthermore, another study demonstrated that IL-6, high-sensitivity CRP, and serum amyloid A were associated with an increased risk of cardiovascular events in healthy individuals and patients diagnosed with vascular diseases. Recently, the CANTOS (Canakinumab Anti-inflammatory Thrombosis Outcome Study) trial showed that the inhibition of the inflammatory cytokine, IL-1β, notably attenuated systemic inflammation, as verified by the serum levels of hs-CRP and IL-6, and reduced the occurrence of recurrent cardiovascular events in patients with myocardial infraction (MI) and residual risk of inflammation [27]. Emerging evidence suggests that IL-1β plays a crucial role in the development of atherosclerosis and MI [28,29]. This cytokine is recognized as being pivotal in instigating the release of other cytokines and chemokines that contribute to inflammatory responses. The focus on mitigating IL-1β-induced inflammation has gained significant interest in the domains of cardiovascular pharmacology, biology, and medicine.

Accumulating evidence suggests that IL-1β secretion is predominantly governed by inflammasomes, which are a complex of multiple intracellular proteins that function as a molecular platform to activate the cysteine protease, caspase-1 [30,31]. Caspase-1 is recognized as an enzyme responsible for converting IL-1β (ICE), so its activation is key to regulating the release of IL-1β and subsequent inflammation. The formation of the inflammasome is triggered by harmful stimuli derived from pathogens, dead or damaged cells, and irritants, such as tissue injury, autoantigens termed damage-associated molecular patterns (DAMPs), and signals from exogenous pathogens (known as pathogen-associated molecular patterns (PAMPs) [32,33]. Bioactive signals trigger an innate immune response, which contains and neutralizes the targeted threat, facilitating the healing process. However, if the inflammatory response persists beyond the original threat, it can lead to chronic inflammation that causes adverse tissue remodeling and diseases. The above PAMPs and DAMPs can be identified by germline-encoded pattern recognition receptors (PRRs), which are mainly expressed in the cells of the innate immune system, including monocytes, neutrophils, and dendritic cells (DCs). It has been reported that several types of PRRs are involved in the formation of inflammasome assembly, such as the nucleotide-binding oligomerization domain-like receptor (NLR) family pyrin domain-containing 1 (NLRP1), NLRP3, NLR family caspase-recruitment domain-containing 4 (NLRC4), absent in melanoma 2 (AIM2), and pyrin [30]. However, NLRP3 is unique in recognizing a wide range of stimuli, more particularly DAMPs, and is involved in the pathogenesis of sterile inflammatory diseases [4,5]. Lastly, there has been accumulating studies concerning the association between infection and ACS in terms of the involvement of several pathogens. The severity of infection is notably associated with an increased risk of ACS, possibly due to the host’s response to infection. However, experimental verification data supporting the role of acute infection on atherosclerosis progression are lacking. Emerging evidence has suggested that the activation of lesion-resident inflammatory cells could be triggered by circulating cytokines, released during acute infection. Undoubtedly, the above mechanism could be involved in advanced COVID-19, which is characterized by excessive cytokine production and multiorgan system failure [34].

## 2. NLRP3 Inflammasome and Atherosclerosis

Over 2500 research articles and reviews have been published since last year concerning inflammasome. The NLRP3 inflammasome is commonly expressed in several types of immune cells, including monocytes, macrophages, dendritic cells, and smooth muscle cells, which are the mesangial cells of coronary arteries [35,36,37,38]. However, excessive lipid deposition can activate the NLRP3 inflammatory response, leading to plaque necrosis [39]. In foam cells and macrophages, the NLRP3 inflammatory vesicles are mainly found in the cytoplasm and are associated with the crystallization of intracellular and extracellular cholesterol crystals [40]. A previous study demonstrated that ox-LDL treatment enhanced nuclear receptor subfamily 3 group C member 2 (NR3C2) levels, an inflammation-related factor, which interacts with NLRP3, and promoted apoptosis and inflammation in human coronary artery endothelial cells (HCAECs). By contrast, NR3C2 silencing inhibited ox-LDL-induced inflammation and apoptosis in these cells. The aforementioned study supported the significant role of NR3C2 and NLRP3 in ox-LDL-induced inflammation in HCAECs and further highlighted their values in the inflammatory response in CAD [41]. NLRP3 activation in macrophages is a critical mechanism driving atherosclerotic inflammation, which ultimately leads to the development of atherosclerosis [42,43]. The NLRP3 inflammasome is recognized as a significant contributor to the molecular pathology of atherosclerosis, as evidenced by various studies in animal models and atherosclerotic patients [44,45,46,47]. These studies suggested that NLRP3 inflammasome activation can lead to the production of IL-1β and IL-18, which promote atherosclerotic plaque progression and instability [48]. In a previous study by Wan et al. [49], the authors used the NLRP3 silencing technology to block the activation of the NLRP3 inflammasome and downregulate ICAM-1 and VCAM-1 in the intima, thus attenuating atherosclerosis and stabilizing atherosclerotic plaques in a diabetic atherosclerosis in vivo model. Additionally, Zheng et al. [50] showed that NLRP3 silencing delayed atherosclerosis progression via decreasing the plaque content of macrophages and enhancing that of smooth muscle cells. Another study also found that the expression of NLRP3 inflammasome-related genes was markedly higher in human atherosclerotic plaques compared with nonatherosclerotic vessels. More specifically, the expression of the NLRP3 inflammasome-related genes was higher in patients with symptomatic lesions [51,52]. Overexpression of NLRP3 in the aorta is also associated with a higher risk for the development of CAD [53]. Other studies demonstrated that the levels of NLRP3, ASC, caspase-1, IL-1β, and IL-18 were differentially upregulated in unstable carotid atherosclerotic plaques in patients undergoing carotid endarterectomy [40,52,54]. The activation of NLRP3 inflammasomes is triggered by multiple stimuli, such as mitochondrial dysfunction, reactive oxygen species production, ion flux, and lysosomal damage [30,55,56]. The mechanisms by which NLRP3 inflammasomes are activated are intricate and not yet completely understood regarding how NLRP3 responds to these signals and initiates the formation of the NLRP3 inflammasome [30].

## 3. Major Inflammation Modulators of NLRP3 Inflammasome

Numerous studies indicated that the interaction between NLRP3 and ligands induces the assembly of inflammasomes, which in turn activates caspase-1, leading to the processing and release of mature IL-1β and IL-18 [52,57,58]. Both IL-1β and IL-18 play crucial roles in the development of atherosclerosis [59].

Recent research has identified IL-1β and IL-18 as the most important inflammatory cytokines that promote atherosclerosis development [60]. IL-1β is primarily expressed in myeloid cells, including macrophages and DCs, and in other cell types, such as endothelial cells and fibroblast. IL-1β acts via binding to its receptor (IL-1 receptor type 1, IL-1R1), thus recruiting IL-1R3 to form ternary complexes, which in turn recruit MyD88. The above process eventually results in the activation of the NF-κB and mitogen-activated protein kinase (MAPK) signaling pathways. Since IL-1R2 lacks a cytoplasmatic domain, it acts as a decoy receptor. IL-1β promotes the onset of an inflammatory phenotype in endothelial cells and VSMCs, characterized by the expression of the adhesion molecules ICAM-1 and VCAM; inflammatory cytokines and chemokines such as IL-6, MCP-1, and Il-8; and matrix metalloproteinases (MMPs), [61,62,63] thus promoting the accumulation of macrophages. Emerging evidence has suggested that IL-1β is involved in atherogenesis, where it acts as a soluble mediator on tissues and organs at a distance [64,65]. IL-1β was downregulated or absent in the blood mononuclear cells of healthy subjects, and it was notably upregulated in diseased patients [66]. Furthermore, IL-1β silencing in ApoE-/- mice significantly attenuated the formation of atherotic plaque [67]. In mice prone to atherosclerosis, plaque progression was reduced following Il-1R ablation [68]. Moreover, it stimulates the secretion of a range of other cytokines and induces the production of endothelin-1 and adherence molecules in the endothelium, thus facilitating leukocyte migration, maintaining the cycle of inflammation, and inducing the generation of nitric oxide in VSMCs [69,70,71,72].

IL-18 is mainly expressed in macrophages, while its receptor, a/b, is expressed in various types of cells, including macrophages, endothelial cells, and VSMCs [73,74]. While the IL-18-binding protein acts as a soluble decoy receptor, similar to IL-1R2, IL-18 is constitutively expressed and requires caspase-1 cleavage to become biologically active [28,75]. Mature IL-18 forms ternary complexes with IL-18Ra and IL-18Rb, activating IRAK/TRAF6, NF-κB, and MAPK pathways through MyD88 recruitment [76]. While IL-18 can induce the expression of adhesion molecules, inflammatory cytokines, chemokines, and MMPs, its inflammatory effect is less than that of IL-1β. Notably, IL-18 synergizes with IL-12, thus inducing the secretion of interferon-γ (IFN-γ) from Th1 cells, NK cells, macrophages, DCs, and VSMCs, but not from endothelial cells [74,76]. Therefore, it is considered that both IL-1β and IL-18 act as proinflammatory cytokines. The significant effect of IL-18 on atherosclerosis has been widely investigated. Therefore, it has been reported that IL-18 is associated with IL-1β both biologically and structurally. IL-18 binding protein [77] is an IL-18-specific inhibitor, which is largely derived from endothelial cells and monocytes. Structurally, IL-18BP is composed of an immunoglobulin domain [78]. IL-18BP can also act as a natural inhibitor, since it binds with mature IL-18, but not with pro-IL-18, with high affinity, thus interacting with cell surface receptors [79]. The range of circulating IL-18BP levels in healthy individuals is 0.5–7 ng/mL. However, higher levels of IL-18BP have been observed in various autoimmune or inflammatory disorders [80,81,82]. Previous studies revealed that IL-18BR overexpression could prevent the formation of fatty streaks in the thoracic aortas of ApoE knockdown mice, thus attenuating the progression of atherosclerotic plaques. IL-18BP has a strong affinity for IL-18 and acts as an essential regulator of immune and inflammatory responses in IL-18-associated diseases [57]. In an ApoE-/- mouse model, IL-18 could stimulate atherogenesis via an IFN-γ-dependent manner [83]. Furthermore, atherosclerotic lesions were smaller in IL-18 gene-deficient mice [84]. Conversely, IL-18-deficient ApoE-/- mice exhibit a decrease in the extension of atherosclerotic plaques [85]. In addition, another study demonstrated that IL-18 variants could affect the clinical outcomes in patients with CAD [86]. Overexpression of IL-18BP and IL-18 silencing in ApoE-/- mice could inhibit IL-18 activity in vivo, eventually promoting atherosclerotic injury [29]. Another study showed that IL-18R-/- mice exhibited increased body weight, ectopic lipid accumulation, enhanced inflammation, and diminished AMPK signaling pathways in skeletal muscles [87]. Additionally, IL-18 levels were notably enhanced in obese patients and patients with type 2 diabetes [88,89]. IL-18, a costimulatory cytokine that mediates adaptive immunity, is required for the production of IFN-γ [90].

## 4. NLRP3 Inflammasome and Pyroptosis

The activation of the NLRP3 inflammasome results in pyroptosis, a regulated cell death mechanism [91,92]. In 2015, the protein gasdermin D (GSDMD) was discovered to be responsible for executing pyroptosis [92,93,94]. In addition to cleaving the precursors of IL-1β and IL-18, active caspase-1 cleaves GSDMD into two domains, namely, the N-terminal domain (GSDMD-C, 22kD) and the C-terminal domain (GSDMD-C, 22kD). After being cleaved, GSDMD-N translocates into the inner leaflet of the plasma membrane, where it binds phosphoinositides to form oligomeric pores with an inner diameter of 10–20 nm. The above process results in pyroptotic death. On the other hand, GSDMD-C normally prevents pyroptosis via inhibiting GSDMD-N under resting conditions. The pores formed by GSDMD can disrupt the osmotic potential, thus promoting water influx, cell swelling, and eventually cell lysis. The release of bioactive IL-1β and IL-18, along with intracellular DAMPs, including S100 and high mobility group box-1, causes inflammatory responses. Since there is a lack of secretion signals of the above two cytokines, their underlying secretion mechanism remains unknown. However, it has been recently reported that both IL-1β and IL-18 are released outside the cells via pores formed by GSDMD. In contrast with caspase-1, murine caspase-11, which is also bound and activated by oxidized phospholipids [95], can directly bind to cytosolic lipopolysaccharides, thus promoting the cleavage of GSDMD and pyroptosis [94,96,97]. However, caspase-11/4/5 cannot directly cleave the precursor of IL-1β or IL-18, while the caspase-11/4/5-induced GSDMD-forming pores lead to Kþ efflux, thus inducing NLRP3 inflammasome activation [98]. Recent studies also suggested that GSDME could be also involved in pyroptosis [99,100]. Therefore, pyroptosis is now considered as GSDM-mediated necrotic death [101].

## 5. Role of Hydrogen Sulfide in Endothelial Dysfunction

Another therapeutic targeted and promising modulator of inflammation is hydrogen sulfide. H2S is an identified and recognized gasotransmitter after nitric oxide and carbon oxide. H2S is produced by homocysteine trans-sulfide metabolism as an endogenous methionine catalysis product. There are four major synthetases of H2S: cystathionine synthase (CBS), cystathionine lyase (CSE), cysteine aminotransferase (CAT), and 3-mercaptopyruvate sulfur transferase (3-MST). CSE and 3-MST are largely expressed in cardiovascular tissues, while CBS is much less prevalent [102,103]. Although it has long been considered a toxic gas, recent studies uncovered its fundamental role in cardiovascular homeostasis. Therefore, its deficiency has been associated with several cardiovascular diseases [104]. Furthermore, H2S has also emerged as a critical molecule for controlling endothelial function at home, and impairment of its endogenous production is associated with ED pathogenesis [105]. Numerous studies have shown that H2S acts as a vasculoprotective gasotransmitter by modulating various cellular pathways and interfering with a range of vascular diseases. It inhibits atherogenic changes in low-density lipoproteins (LDL) and monocyte adhesion to endothelial cells (ECs) that occur due to activation, promotes vasorelaxation, and prevents intimal hyperplasia by blocking the migration and proliferation of VSMCs. H2S also inhibits vascular calcification, thrombogenesis platelet aggregation, and macrophage foam cell formation and degranulation. Moreover, it could reduce inflammatory responses and the plasma levels of homocysteine (Hcy) in vivo [106,107,108,109,110,111,112]. In specific cases, CSE deletion from the endothelium was associated with endothelial inflammation and atherosclerosis, which was then reversed by polysulfide donors [113,114]. H2S treatment could reduce the production of inflammatory mediators, such as VCAM-1, ICAM-1, and MCP-1, in TNF-α-induced endothelial cells [115]. The primary mechanism underlying this protective effect is the inhibition of soluble TNF-α shedding and its associated MCP-1 release. Additionally, in endothelial cells, exogenous H2S could inhibit the NF-κB signaling pathway and suppress the angiotensin II-induced inflammatory responses [116]. In addition to inhibiting the NF-κB pathway, H2S also attenuates pulmonary endothelial cell inflammation and subsequent pulmonary hypertension [117]. Endogenous H2S could directly induce sirtuin1 (SIRT1) sulfhydration and stability, thus reducing aortic inflammation and atherosclerotic plaque formation [118]. CSE deficiency increases endogenous sulfur dioxide (SO2) levels in endothelial cells. However, the inhibition of endogenous SO2 could exacerbate the CSE knockdown-induced NF-κB signaling pathway and the release of its downstream inflammatory factors in endothelial cells. The above findings suggest that the increased endogenous SO2 generation could probably act as a compensatory mechanism for the downregulated CSE/H2S pathway in endothelial inflammatory responses [119]. According to the study, the anti-inflammatory effects of H2S donors may be beneficial for the treatment of endothelial inflammation-related cardiovascular disease.

## 6. Contemporary Research

### Potential Anti-Inflammatory Therapies Tested in Clinical Studies

**Figure 1:**
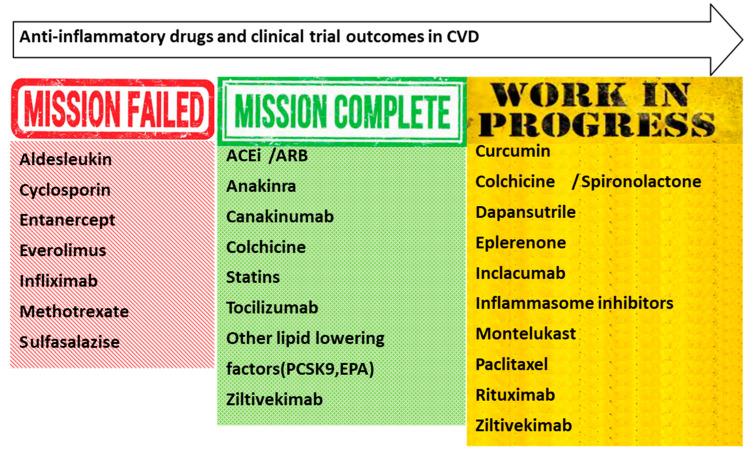
Anti-inflammatory drug candidates for the prevention and treatment of CVD.

CANTOS, a randomized, double-blind, placebo-controlled trial, ended in 2017 and suggested that canakinumab, a human monoclonal antibody against IL-1β, could improve cardiovascular outcomes (Figure 1). Therefore, it was the first clinical trial in history, which validated the inflammatory hypothesis. Canakinumab proved effective, for statistical significance at a dosage of 150 mg, at preventing adverse cardiac events over a median of 3.7 years among patients with a history of MI and increased hsCRP. It was also effective at reducing hsCRP in a dose-response manner. Moreover, canakinumab, due to its lowering of hsCRP levels, has been proven to be most effective in the subgroup of chronic kidney disease. A significant determinative of recurrent CVR is residual inflammatory risk. On the other hand, it was implicated with an increased risk of fatal infection or sepsis, even when patients with chronic/recurrent infection were excluded [27]. Another key clinical trial was COLCOT (Colchicine Cardiovascular Outcomes Trial), whose outcomes were published in 2019 and which depicted that the subgroup of a low dose of 0.5 mg of colchicine demonstrated a significant reduction in adverse CV events post-MI [120]. The study population consisted of patients with recent MI, randomized to either placebo or colchicine plus the optimal medical therapy according to the guidelines. These patients were followed for a median of 22.6 months, and the study results concluded that in the colchicine-treated group, there was a reduction in the composite primary outcome of 23% compared with placebo, especially contributing to the reduction in stroke and urgent revascularization. The LoDoCo2 randomized clinical trial (Low-Dose Colchicine for Secondary Prevention of Cardiovascular Disease 2) further enhanced the results from COLCOT, demonstrating that the anti-inflammatory drug colchicine reduces the risk of CV events in patients after recent MI and with stable CAD, respectively [121]. The molecule of colchicine disrupts tubulin and, as a result, reduces the migration and replication of inflammatory cells. This action, among others that are not well defined yet, affects endothelial function and inhibits the NLRP3 inflammasome [122]. Another action is the indirect reduction of the activation of IL-1β and downstream in IL-6 and CRP, which are well-known mediators that activate macrophages and propagate atherosclerosis [122]. According to these results, colchicine has received a Class IIb recommendation at the ESC guidelines on cardiovascular disease prevention in clinical practice: for the prevention of recurrent CV events in the group of high-risk patients. On the other hand, as far as the adverse events are concerned, the increase in non-CV and all-cause mortality in the LoDoCo2 trial is still being debated. Results that are in contrast with the long-term data from familial Mediterranean fever patients from Ben-Chetrit et al. and a comprehensive meta-analysis of 14,188 patients from 21 randomized controlled trials, published in 2021, proved to be very reassuring for the opposite [123,124]. Finally, TNF-α inhibitors have been arguable in terms of preventing MACEs. Ahlehoff et al. and Jacobsson et al. showed that in diseases such as psoriasis or rheumatoid arthritis, the incidence of hard CV outcomes was lower with TNF-α inhibitor therapy [125,126]. On the other hand, a respectable number of clinical trials depicted disappointing results as far as inflammation mediators were concerned. Among TNF-α inhibitors, methotrexate, or ustekinumab, and in the ATTACH trial with infliximab, the rate of major adverse CV events did not differ, and there was neither a decrease in NYHA III–IV HF patients’ status nor a reduction of mortality and hospitalization events [127,128]. A renewal trial with etanercept, a TNF soluble antagonist, showed no clinically relevant benefit in congestive heart failure. CLEVER-ACS demonstrated no reduction in MI size and improvement of microvascular obstruction in STEMI patients treated with everolimus. Lastly, neither did the TETHYS trial show that methotrexate reduced infarction size and, on the contrary, worsened LVEF at 3 months, and the ALL HEART trial that studied patients aged 60 years or older with CAD but no history of gout demonstrated no difference in the primary outcome of nonfatal MI, nonfatal stroke, or cardiovascular death between participants randomized to allopurinol therapy and those randomized to standard guideline therapy. Clearly, more evidence is required to assess the role of TNF-α inhibitors in CAD. The major trials that have addressed the impact of various anti-inflammatory medications on cardiovascular outcomes are depicted in Table 1.

## 7. The Role of Inflammatory Mediators in Cardiovascular Disease

Nowadays, there are several studies that examine the inflammatory processes and their mediators that cause ED, focusing on key representatives, proteins such as ICAM-1, VCAM-1, and E, P-selectin. ICAM-1 and VCAM-1 overexpression promotes ED, which is an early stage of atherosclerosis. Additionally, the upregulation of these molecules is one of the hallmark characteristics of ED [159,160]. Furthermore, previous studies also demonstrated that atherosclerosis was associated with intercellular monocyte adherence upregulation to the endothelium. Although ICAM-1, VCAM-1, and E-selectin play a significant role in the migration of lymphocytes, their prevalence in the case of atherosclerotic plaques is far from equal. From the above, only VCAM-1 plays a major part in both the early and the later stages of atherosclerosis. As a consequence, it provides a suitable and easy target for diagnosing pathological situations [161,162,163]. Another modulator associated with cardiovascular disease is PTX3. It modulates inflammatory cells, thus stimulating vascular inflammation, and is synthesized and secreted by smooth muscle cells, vascular endothelial cells, monocytes, macrophages, and fibroblasts in response to TNF-α, IL-1, and lipopolysaccharides [164,165,166]. Increased levels of PTX3 reflect an increased vascular inflammatory status due to its direct synthesis by cells that are involved in atherosclerosis. As a result, it could represent a potent marker for the development and prediction of atherosclerosis CV diseases [167,168]. Another recent study that further enhances its key role suggests that PTX3 might be of significant interest for studies in the near future as a potential predictor of long-term mortality in STEMI patients. Furthermore, the design of prospective studies based on the current knowledge in this area would be of great interest [169].

The urokinase plasminogen activator (uPA) and its receptor (uPAR), a fibrinolytic factor, has been associated with inflammatory response, vascular homeostasis, and an immune homeostasis system [170]. Additionally, a previous study by Mendelsohn et al. showed that chimeric antigen receptor T cells targeting uPAR-expressing senescent cells in atherosclerotic plaques and fibrotic livers could remove these cells and improve glucose metabolism. Furthermore, a novel vaccine targeting CD153-expressing senescent T cells could also improve metabolism in obese mice, while it could diminish atherosclerotic plaques in an atherosclerosis mouse model [171].

VWF, a large multimeric glycoprotein involved in hemostasis and local inflammatory responses, serves a key role in the adhesion of platelets to the subendothelium of the impaired endothelial layer of stenotic arteries. VWF dysfunction has been associated with the development of CAD and its complications. Therefore, due to its prominent role in arterial thrombosis, targeting the interaction between VWF with the vessel wall and platelets could be considered a significant approach for preventing CAD [172].

Endocan, a well-studied molecule, also known as endothelial-cell-specific molecule-1, is constitutively expressed from human endothelial cells of the lungs and kidneys [173]. It has been reported that endocan is regulated by vascular endothelial growth factor, and it is considered a significant marker of sepsis and cancer. Inflammatory cytokines and pro-angiogenic growth factors commonly upregulate endocan [174].

Finally, due to parallel increased interest as far as COVID-19 and endothelial damage are concerned, it is expected that even more clinical drug trials will be initiated in the near future. Emerging evidence has suggested that COVID-19 is an endothelial disease. Therefore, it was hypothesized that inflammation, cytokine storm, oxidative stress, and coagulopathy could be caused by endotheliopathy. However, several patients with endothelial dysfunction, associated with various comorbidities, such as hypertension, diabetes, and obesity, develop more severe forms of COVID-19, possibly due to the additional changes of the already-impaired vascular endothelium [3]. The correlation of inflammatory molecules with cardiovascular disease and their outcome has been tested in a variety of clinical trials (Table 2).

## 8. Conclusions

There are a plethora of articles, reviews, and clinical trials dedicated to exploring inflammation modulators/biomarkers and pro-/anti-inflammatory (such as IL-1, IL-6, IL-18, TNF-α and CRP, PTX3, and adhesion molecules) and signaling pathways (such as NLRP3 inflammasome, NF-κB), which are implicated in endothelium dysfunction and, as a result, in atherosclerosis. A better understanding of all these key players in inflammation and endothelial function and the development of specific treatments/inhibitors, without compromising the immune system’s defense against pathogens, will not only offer new therapeutic modalities but also contribute to the pathogenesis of atherosclerosis and other cardiovascular diseases.

## Figures and Tables

**Table 1:** Clinical studies of treatments targeting inflammation in the context of CVDs.

Trial	Drug	Target Molecule-Signaling Pathways	Study Population Primary Outcome	Refs.
Cantos	Canakinumab	IL-1β inhibition	Previous MI, nonfatal MI, nonfatal stroke, CV death, reduced hsCRP, IL-6−17% in primary endpoints/higher incidence of fatal infections	[27]
ATTACH	Infliximab	NF-κB/TNF-α	Chronic heart failure ↑ mortality and hospitalization/no improvement in the clinical status of NYHA III–IV HF patients	[129]
Sulfasalazine and Endothelial Function (NCT00554203)	Sulfasalazine	↓ NF-κB activation ↓ inflammatory TNF-α-induced genes	History of CAD/no amelioration of ED in patients with CAD, no effects on systemic inflammatory biomarkers	[34]
RENEWAL trial	Etanercept	TNF-α soluble antagonist	No clinically relevant benefit in congestive heart failure	[130]
VCUART3 (NCT01950299)	Anakinra	IL-1R blocker	STEMI patients/ lower incidence of death or new-onset heart failure or of death and hospitalization for heart failure/significantly reduces the systemic inflammatory response compared with placebo	[131]
MRC-ILA	Anakinra	As above	NSTEMI/49% reduction in CRP levels over first 7 days/significant increase in MACE at 1-year follow-up	[132]
CLEVER-ACS	Everolimus	mTOR pathway	STEMI PATIENTS No reduction in MI size No improvement of microvascular obstruction	[133]
LILACS	Aldesleukin (recombinant human IL-2)	IL-2 receptor Treg cells	Stable ischemic heart disease and ACS/increase in Tregs but not CD4+ effector T cells	[134]
ASSAIL-MI	Tocilizumab	IL-6	STEMI within 6 h/5.6% increase in myocardial salvage index/no significant difference in infarct size at 6 months/significant reduction in CRP	[135]
RESCUE	Ziltivekimab	IL-6	Chronic kidney disease, CRP ≥2 mg/L/significantly greater reduction in CRP from baseline at 12 weeks, a significant reduction in lipoprotein A, but no change in LDL/HDL ratio	[136]
Study of Dapansutrile Capsules in Heart Failure	Dapansutrile	NLRP3 inflammasome inhibitor	Stable patients with HFrEF/ changes in left ventricular ejection fraction will be analyzed	[137]
NCT04927247	Inclacumab	Monoclonal antibody targeting P-selectin	Sickle cell disease vaso-occlusive crisis/vaso-occlusive pain episode in sickle cell disease	[138]
StratMed-MINOCA	Eplerenone	Reduces blood vessel injury and is used to treat heart failure	MI, AMI with nonobstructive coronary artery myocardial injury-COVID-19 to test the use of eplerenone in patients with heart attack/heart injury who have small vessel disease, including patients with COVID-19	[139]
Curcumin Supplementation Effects on Markers of Cardiovascular Risk, Inflammation, Oxidative Stress, and Functional Capacity in Patients with CAD	Curcumin is produced by turmeric root (*Curcuma longa*)	Promoting the activation of inflammasome (NLPR3)	The effects of curcumin supplementation on inflammatory cytokines	[140]
CIRT	Methotrexate	Replication inhibition of B cells, T cells neutrophils, monocytes	Previous MI and T2 diabetes metabolic syndrome, nonfatal MI, nonfatal stroke, CV death, no change in hs-CRP, IL-6, IL-1β, no reduction in primary endpoints	[141]
TETHYS	Methotrexate	As above	STEMI within 12 h, no effect on creatine kinase release over first 72 h/no difference in CRP levels/significantly worse LVEF in the methotrexate group at 3 months/no effect on the incidence of MACE	[142]
METIS trial	Methotrexate	Ischemic chronic heart failure	No difference in 6 min walk time before vs. after treatment/no effect on CRP levels/no effect on the incidence of MACE	[143]
COLCOT	Colchicine	Inhibition of microtubule polymerization reduced IL-1β, IL-6	1 month after MI, CV death, MI stroke −23% in primary endpoints	[127]
LoDoCo2	Low-dose colchicine	As above	Acute and chronic CAD/as above, −31% of primary endpoints	[121,144]
COLCHICINE-PCI	Colchicine	As above	Patients referred for PCI/no significant difference in PCI-related myocardial injury or MACE within 30 days/elevation of plasma IL-6 and CRP from baseline—24 h s significantly reduced in the colchicine group/increased adverse gastrointestinal symptoms in the colchicine group	[145]
COPS	Colchicine	As above	ACS/no significant difference in MACE/increased total death incidence in the colchicine group, mostly related to sepsis	[146]
COVERT-MI	Colchicine	As above	STEMI within 12 h/no significant reduction in infarct size at 5 days/no significant reduction in MACE at 3 months’ follow-up/no difference in inflammatory marker (WBC count, CRP) at 48 h	[147]
Randomized Trial of Anti-inflammatory Medications and Coronary Endothelial Dysfunction in Patients with Stable Coronary Disease	Colchicine/methotrexate	As above	Patients with stable CAD and either elevated hsCRP or diabetes/metabolic syndrome on stable statin therapy failed to improve coronary endothelial function	[148]
CLEAR SYNERGY	Colchicine/spironolactone	As above	Patients with STEMI/evaluation of markers of neutrophil activity at randomization (baseline) and 3 months’ follow-up in the colchicine versus placebo groups, and examination of clinical and genetic factors that determine the heterogeneity of treatment response and distinguish colchicine responders from nonresponders	[149]
ALL HEART	Allopurinol	Inhibitor of xanthine oxidase, ROS pathway	≥60 years old with ischemic heart disease but no history of gout/no difference in the primary outcome of nonfatal MI, nonfatal stroke, or cardiovascular death	[150]
CIRCUS	Cyclosporine	T cell activation, macrophage ROS/cytokine production	STEMI within 12 h/no effect on any cause of death/No effect on remodeling or MACE incidence at 6 months	[151]
CYCLE	Cyclosporine	As above	STEMI within 6 h/no effect on ST-segment resolution at 1 h/no effect on LV remodeling or incidence of MACE at 6 months	[152]
Aortic Stenosis Progression Observation: Measuring Effects of Rosuvastatin [153]	Rosuvastatin	Inhibits NF-κB, TNF-α, and IL-6	Intensive lipid lowering using rosuvastatin 40 mg daily on the progression of AS/↓ CRP levels ↓ LDL cholesterol	[153]
GISSI-HF (Gruppo Italiano Per Lo Studio Della Sopravvivenza Nell’Insufficienza Cardiaca)	Rosuvastatin	As above	Heart failure/↓ CRP levels ↓ LDL cholesterol	[154]
CORONA	Rosuvastatin	As above	Heart failure/↓ CRP levels ↓ LDL cholesterol	[155]
anaRITA MI2	Rituximab	B-cell depletion with CD20	Patients with STEMI and LVEF at 6 months with CMR	[156]
Air Pollution (PM2.5) on Accelerated Atherosclerosis: A Montelukast Interventional Study in Modernizing China	Montelukast	Leukotriene receptor antagonist	Subclinical atherosclerosis defined as changes in brachial flow-mediated dilation and carotid intima–media thickness	[157]
PAC-MAN	Paclitaxel	Blocks cellular proliferation (antimicrotubule agents)	Patients with stable CAD/reduction in plaque size measured by CCTA from baseline to 6–8 months	[158]

ACS: acute coronary syndrome; AS: aortic stenosis; CAD: coronary artery disease; CCTA: coronary computed tomography angiography; CMR: cardiac magnetic resonance; CV: cardiovascular; CVRF: CV risk factors; CRP: C-reactive protein; ED: endothelial dysfunction; HDL: high-density lipoprotein; HFrEF: heart failure reduced ejection fraction; IL: interleukin; IMT: intima–media thickness; LDL: low-density lipoprotein; LVEF: left ventricular ejection fraction; MACE: major adverse cardiovascular event; MI: myocardial infarct; NF-κB: nuclear factor kappa B cells; NSTEMI: non-ST-segment elevation myocardial infarction; NYHA: New York Heart Association; PAD: peripheral artery disease; PCI: percutaneous coronary intervention; ROS: reactive oxidative stress; STEMI: ST-segment elevation myocardial infarction; TNF-α: tumor necrosis factor-α; WBC: white blood cells.

**Table 2:** Inflammation modulators under clinical trials.

Target Molecules	Signaling Pathways	CVD Primary Endpoint Outcome	Refs.
ICAM-1 (CD54)	Leukocyte adhesion	Associates with the incidence of CAD and carotid atherosclerosis independent of known CVRF.	[163]
VCAM-1 (CD106)	Leukocyte adhesion	Baseline VCAM-1 is increased in initially healthy middle-aged men who develop cardiovascular disease.	[163]
E-selectin (CD62E)	Leukocyte adhesion	Associates with the incidence of CAD and carotid atherosclerosis independent of known CVRF.	[175]
P-selectin (CD62)	Leukocyte adhesion	Elevated levels predict early adverse events in patients with presumed CAD.	[175]
Correlation of atherosclerotic plaque volume and intima–media thickness with soluble P-selectin	Leukocyte adhesion	Correlation between P-selectin and the progression of atherosclerosis as measured by plaque volume and IMT in the carotid and femoral arteries, respectively. Secondary endpoints will include the correlation of established (hypertension, smoking, diabetes, dyslipidemia) and novel risk factors (hs-CRP, P-selectin, CETP, ICAM-1.	[176]
MCP-1	Leukocyte adhesion	Correlation with the risk of incident PAD and CAD independent of traditional cardiovascular risk factors.	[177]
Vascular endothelial receptor activity in patients with CAD on medication with statins/MCP-1	Leukocyte adhesion	MCP-1-induced monocyte chemotaxis after 1-month treatment with atorvastatin 40 mg or a placebo once a day.	[178]
PTX3/Lipid Assessment Trial Italian Network (LATIN)	NF-κB	Patients with MI with ST elevation PTX3 prognostic tool: 3-month mortality in patients with MI and ST elevation.	[27,179]
Dynamic changes in pentraxin 3 and neprilysin in ST-segment elevation myocardial infarction	PTX3 and neurolysin	Confirmation of the differences in kinetics between the two pentraxins CRP and PTX3, with PTX3 levels being high already in the acute samples while the peak for CRP came 1–3 days after PCI. Neprilysin is not generally elevated during STEMI, although a few patients showed very high levels.	[169]

CAD: coronary artery disease; CETP cholesteryl ester transfer protein CVRF: CV risk factors; hs-CRP: high-sensitivity C-reactive protein; ICAM-1: intracellular adhesion molecule-1; IL: interleukin; IMT: intima–media thickness; MCP-1: monocyte chemoattractant protein-1; MI: myocardial infarct; NF-κB: nuclear factor kappa B cells; PAD: peripheral artery disease; PCI: percutaneous coronary intervention; PTX3: pentraxin-related protein 3; STEMI: ST-segment elevation myocardial infarction; VCAM-1: vascular cell adhesion molecule-1.

## Data Availability

Not applicable.
